# TEAD4 overexpression promotes epithelial-mesenchymal transition and associates with aggressiveness and adverse prognosis in head neck squamous cell carcinoma

**DOI:** 10.1186/s12935-018-0675-z

**Published:** 2018-11-12

**Authors:** Wei Zhang, Jin Li, Yaping Wu, Han Ge, Yue Song, Dongmiao Wang, Hua Yuan, Hongbing Jiang, Yanling Wang, Jie Cheng

**Affiliations:** 1 Jiangsu Key Laboratory of Oral Disease, Nanjing Medical University, 136 Hanzhong Road, Jiangsu, 210029 People’s Republic of China; 2 Department of Oral and Maxillofacial Surgery, Affiliated Stomatological Hospital, Nanjing Medical University, 136 Hanzhong Road, Nanjing, 210029 People’s Republic of China

**Keywords:** Head and neck squamous cell carcinoma, Hippo signaling, TEAD4, EMT, Prognostic biomarker

## Abstract

**Background:**

Deregulated Hippo signaling has been uncovered to be intricately involved in tumorigenesis. Transcriptional factor TEADs serve as key mediators of Hippo signaling and have been increasingly appreciated as putative oncogenes driving cancer initiation and progression. However, its expression pattern and oncogenic role of TEAD4 in head and neck squamous cell carcinoma (HNSCC) remain largely unexplored.

**Methods:**

TEAD4 mRNA expression in HNSCC was determined by data mining and analyses from TCGA dataset and four independent cohorts with transcriptional profiling data publically available. The protein abundance of TEAD4 was measured by immunohistochemistry in 105 primary HNSCC samples and associations between its expression and clinicopathological parameters and patient survival were evaluated. The oncogenic roles of TEAD4 was further determined by 4-nitroquinoline 1-oxide (4NQO)-induced animal model, both knockdown/overexpression assay and TGF-β1-induced epithelia-mesenchymal transition (EMT) in vitro.

**Results:**

Both mRNA and protein abundance of TEAD4 were significantly increased in HNSCC as compared to its non-tumor counterparts. Overexpression of TEAD4 significantly associated with high pathological grade, cervical node metastasis, advanced clinical stage and reduced overall and disease-free survival. In the 4NQO-induced HNSCC mouse model, increased TEAD4 immunostaining was found associated with disease progression. TEAD4 knockdown significantly inhibited cell proliferation, migration and invasion, and induced cell apoptosis in HNSCC cells, while its overexpression resulted in opposite effects and EMT. Moreover, TEAD4 was critically involved in TGF-β1-induced EMT in HNSCC cells.

**Conclusions:**

Our findings reveal that TEAD4 serves as a novel prognostic biomarker and putative oncogene for HNSCC by promoting cell proliferation, migration and invasion, and EMT.

**Electronic supplementary material:**

The online version of this article (10.1186/s12935-018-0675-z) contains supplementary material, which is available to authorized users.

## Background

Head and neck squamous cell carcinoma (HNSCC) is the sixth common cancer worldwide with more than 350,000 cancer-related deaths per year. Multiple etiological factors have been identified to critically contribute to this malignancy including smoking abuse, alcohol consumption, betel quid chew and human papillomavirus (HPV) infection [1]. Even though combined and multidisciplinary therapy against HNSCC have been established, the long-term survival rate of HNSCC patients has not been markedly improved in the past decades [2]. Major prognostic factors include invasive depth, cervical lymph node metastasis and advanced TNM stage [3]. Much efforts have been made to unveil the cellular and molecular mechanisms of HNSCC tumorigenesis [4]. However, the precise mechanisms under its initiation and development still remain fragmented. Thus, identification of new biomarkers and therapeutic targets for HNSCC is urgently needed for clinicians to improve the patients’ prognosis.

The Hippo signaling pathway has increasingly been recognized as a key and indispensable mediator in tissue homeostasis, organ size control, metabolism, regeneration and tumorigenesis [5]. Defects in Hippo signaling and hyperactivation of its downstream effectors yes-associated protein (YAP) and transcriptional coactivator with PDZ-binding motif (TAZ) essentially contribute to cancer initiation, outgrowth, metastatic dissemination and therapeutic resistance [6, 7]. Pervasively activated YAP and TAZ in human malignancies accumulated in the nucleus where they drive gene transcription mainly by forming complexes with TEA domain DNA-binding family of transcription factors (TEADs) [8]. In mammal, there are four TEAD protein members, namely TEAD1–4. TEADs are broadly expressed but each member has tissue-specific expression pattern which suggests tissue-specific roles for each TEAD [9]. Previous studies have revealed important functions of TEAD members in various biological processed and human diseases [10, 11]. TEAD transcription factors are not only crucial for developmental process, but also play important roles in tumor initiation and progression [9, 11, 12]. TEADs promote cell proliferation, migration and invasion, epithelial-mesenchymal transition in several solid tumors including prostate, breast, colorectal and gastric cancers by binding with or without YAP/TAZ [13–15]. Previous reports largely focused on the expression and biological roles of YAP and TAZ during tumorigenesis [7, 16, 17]. However, the accurate biological functions of TEADs in human cancer are just beginning to disclose in selected contexts and remain yet unexplored in HNSCC.

Here, we sought to determine the expression of TEAD4 and its clinicopathological significance in human HNSCC samples and chemical-induced HNSCC animal model. Moreover, we determined the tumorigenic roles of TEAD4 by functional assays in vitro and revealed the critical links between TEAD4 and EMT in HNSCC.

## Materials and methods

### Patients and tissue specimens

A total number of 105 patients with primary HNSCC (Jan. 2008 and Dec. 2014.) receiving radical resection of cancer at the Department of oral and maxillofacial surgery, Nanjing Medical University were enrolled. Written informed consent was obtained from these patients. Patient inclusion criteria were described as follows: (1) primary HNSCC with no prior chemotherapy or radiotherapy; (2) patients underwent radical tumor resection and neck lymph node dissection; (3) detailed demographic, clinical, pathological and follow-up data available. The archived tissue samples and haematoxylin–eosin stained sections of each patient were retrieved. The previous histological diagnosis as SCC were further histopathologically conformed according to the established histological criteria. Twenty samples of healthy oral mucosa were obtained from intraoral trauma surgery at the same period. This study protocol was reviewed and approved by the Research Ethic Committee of Nanjing Medical University.

### Cell lines and chemicals

A panel of HNSCC cell lines including Cal27, Fadu, SCC4, SCC25, HN4 and HN6 were used. Non-tumorigenic HOK and Cal27, Fadu, SCC4 and SCC25 were purchased from American Type Culture Collection (ATCC, Manassas, VA, USA). HN4 and HN6 cell lines were generous gifts from Prof. Wantao Chen from Shanghai Jiaotong University. Cancer cells were grown in DMEN/F12 (Invitrogen) supplemented with 10% FBS (Gibco) and 100 units/ml antibiotics, and maintained at 37 °C. For TGF-β1-induced EMT cell model in vitro, morphological changes and relevant markers expression were monitored in cells which were treated with recombinant human TGF-β1 (rhTGF-β1, 10 ng/ml, R&D Systems) for indicated times.

### Small interference RNA (siRNA) DNA constructs and transfection

The siRNA oligonucleotides including TEAD4 siRNA-1 (5′-CCGCCAAAUCUAUGACAAATT′, 5′-UUUGUCAUAGAUUUGGCGGTT′) TEAD4 siRNA-2 (5′-CGCUCUGUGAGUACAUGAUTT-3′, 5′-AUCAUGUACUCACAGAGCGTT′) and control siRNA (5′-UUCUCCGAACGUGUCACGUTT-3′, 5′-ACGUGACACGUUCGGAGAATT-3′) were designed and purchased from GenePharma (Shanghai, China). Transfection of siRNA oligonucleotides with final concentration 100 nM was performed with Lipofectamine RNAiMAX (Life Technologies) according to the manufacturer’s instruction. Then cells were harvested for further experiments 48 h after transfection unless otherwise specified.

The human TEAD4 overexpression construct tagged with single FLAG was generated by inserting the TEAD4 full-length cDNA template into plasmid GV141. Following transient transfection with TEAD4 overexpression plasmid, cells were harvested at 48 h for further experiments. Stable cell clones with TEAD4 overexpression were selected by appropriate antibiotics (G418, 500 ng/ml, Sigma) for 2 weeks after plasmid transfection.

### CCK-8 and colony formation assay

Cell proliferation and viability were assessed by absorbance using CCK-8 cell viability assay (Cell Counting Kit-8, Dojindo, Japan) per manufacturer’s instructions. Cells were seeded in 96-well microplates at a density of 2 × 10^3^ cells per well. Cells were incubated in new medium containing 10% CCK-8 reaction solution. After incubation for 2 h, the absorbance was measured on a spectrophotometer microplate reader (Multiskan MK3, Thermo) at a wavelength of 450 nm. Colony formation assay was performed as we previously reported [18].

### Cell apoptosis assessed by flow-cytometric assay

Cells were treated with trypsin (Gbico) and resuspended as single-cell suspension. Cells were stained with Annexin V:PE Apoptosis Detection Kit (BD Bioscience) and submitted to a FACSCalibur flow cytometer (BD Biosciences). Data were analyzed with CellQuest Pro software (BD Biosciences).

### In vitro cell invasion and wound healing assay

For wound-healing assays, cells were seeded at a density of 1 × 10^6^ cell/well in six-well plates. Then we used a sterile 10 μl pipette tip to create an artificial wound on the confluent cell monolayer. The suspended cells were washed thoroughly with PBS, and cells were cultured in medium with 1% FBS (Gibco). The wounds were photographed at 0, 6, 12 and 24 h as indicated. Cell invasion were determined by a Matrigel transwell invasion assay. In brief, 1 × 10^5^ viable cells were suspended in 200 μl of DMEN/F12 (Invitrogen) without serum and seeded into upper chamber precoated with Matrigel (BD Biosciences, USA). Complete medium with 10% serum was added to the lower chamber as chemoattractant. After incubation for 12 h, the non-invading cells were gently removed with a cotton swab, while those invaded cells adherent to the lower side of membrane were stained with a 0.1% crystal violet solution, photographed and counted as our revious reports [19, 20].

### Immunofluorescence assay

For immunofluorescence assays, cells were seeded on glass coverslips 18 h prior to experiment and fixed with 4% paraformaldehyde and washed thoroughly with PBS. After these, the cells were permeabilized in Triton X-100 (0.1% in PBS) for 1 h and washed thoroughly with PBS. Then cells were blocked with 3% bovine serum albumin (BSA) for 30 min at room temperature followed by incubation with primary antibodies against E-cadherin (1:200 dilution) and vimentin (1:150 dilution) overnight, respectively. Cells were further incubated with corresponding secondary antibodies and/or cytoskeleton actin/nuclear staining. Immunofluorescence was visualized under a Zeiss fluorescence microscope or confocal microscope.

### RNA extraction and real time RT-PCR

Total RNA was extracted from cells and subjected to reverse transcription and PCR reactions using PrimeScriptTM RT-PCR kit (Takara) as described previously [19, 20]. Relative mRNA expression was quantified as compared to internal control GAPDH using comparative CT method. The primers were listed as follows: TEAD4 (forward: TCCACGAAGGTCTGCTCTTT, reverse: GTGCTTGAGCTTGTGGATGA) and GAPDH (forward: AGGTGAAGGTCGGAGTCAAC, reverse: AGTTGAGGTCAATGAAGGGG).

### Western blot analysis

Cells were harvested and lysed in ice-clod cell lysis buffer containing protease inhibitor cocktail (Invitrogen). The same amount of protein samples were electrophoresed through 10% SDS-PAGE and transferred to PVDF membranes (Bio-Rad). Following 5% non-fat milk or BSA blocking, these membranes were incubated at 4 °C overnight with primary antibodies TEAD4 (1:1000, ab58310, Abcam), E-cadherin (1:2000, #3195, Cell signaling), N-cadherin (1:1000, #13116, Cell signaling), vimentin (1:2000, #5741, Cell signaling), snail (1:1000, #3879, Cell signaling) and GAPDH (1:2000, sc-32233, Santa Cruz) followed by incubation with horseradish peroxidase(HRP)-conjugated secondary antibodies. Immunoreactive bands on the blots were detected by ECL chemiluminescence kit (Bio-Rad).

### 4-nitroquinoline 1-oxide (4NQO)-induced HNSCC animal model

In the 4NQO-induced HNSCC animal model, squamous cell carcinoma was initiated and progressed in tongue. This experimental was performed as our previous reports with minor modifications [21–23]. In brief, 6-week-old C57BL/6 mice were fed with drinking water containing 50 μg/mL 4NQO for consecutive 16 weeks and then given with normal water for another 8–10 weeks. Animals with normal water was used as controls. Lesions in tongue were visually inspected every week. Samples were harvested at 16, 20 and 24 weeks after chemical administration and subjected to histopathological analyses.

### Immunohistochemical staining and scoring

Immunohistochemical staining for TEAD4 was performed on 4 μm-thick slides from formalin-fixed paraffin-embedded samples using routine procedures as our previously reported [7]. Negative controls without primary TEAD4 antibody (1:200, GTX108750, GeneTex) incubation were included. Immunoreactivity was semi-quantitatively evaluated according to staining intensity and distribution using the immunoreactive score which was calculated as intensity score × proportion score as we reported previously [20, 24]. Intensity score was defined as 0, negative; 1, weak; 2, moderate; 3, strong, while the proportion score was defined as 0, negative; 1, < 10%; 2, 11–50%; 3, 51–80%; 4, > 80% positive cells. The total score ranged from 0 to 12. Accordingly, the immunoreactivity of each slide was categorized into three subgroups based on the final score: 0, negative; 1–4, low expression; 4–12, high expression as we reported before [20, 24].

### Data mining and analysis of TEAD4 mutation and expression in HNSCC via publicly available database

The original data concerning mutational landscape and mRNA expression of TEAD1–4 in HNSCC were retrieved from 3 publicly available databases including cBioPortal (http://www.cbioportal.org/) [25], TCGA (https://cancergenome.nih.gov/) and Oncomine (https://www.oncomine.org/) [26]. TEAD4 mRNA expression levels (log2-transformed) in HNSCC and normal counterparts were retrieved and statistically compared. The associations between expression status of TEAD4 mRNA (high or low using median value as cutoff) and patient survival were determined by Kaplan-Meir analysis.

### Statistical analysis

All quantitative data was presented as mean ± SD from two or three independent experiments and compared with Student’s *t*-test or ANOVA with Bonferroni post hoc test unless otherwise specified. The correlations between TEAD4 expression and various clinicopathological parameters were evaluated by Chi square or Fisher exact test. Patient survival was estimated using Kaplan–Meier method and compared with Log-rank test. The prognostic analyses were performed by univariate and multivariate Cox regression models to determine the individual clinicopathological variables with patient overall survival. *P* values less than 0.05 (two-sided) were considered statistically significant. All statistical analyses were performed using GraphPad Prism 8 or SPSS 21.0 software.

## Results

### Aberrant upregulation of TEAD4 mRNA in HNSCC via bioinformatics analyses

**Fig. 1:**
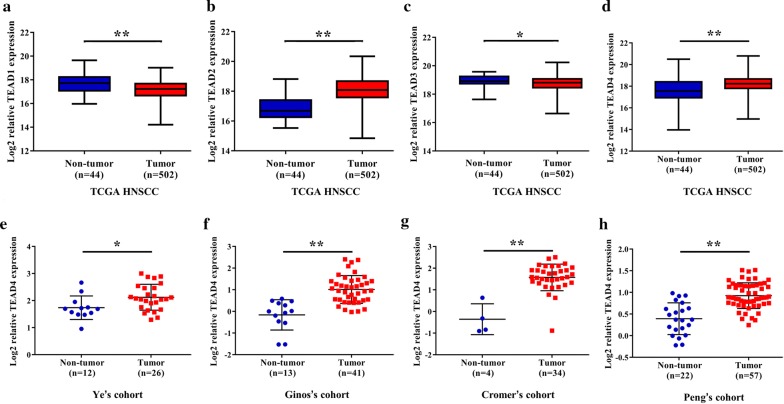
Overexpression of TEAD1-4 mRNA in HNSCC cohorts. The mRNA levels of TEAD1-4 (log2-transformed) were compared between HNSCC samples and normal counterparts in multiple patient cohorts. **a**–**h** The original data were retrieved from Oncomine database and TCGA and then plotted using GraphPad Prism 7 software. Y-axis represents the median intensity, 25th and 75th percentile data. *P < 0.05; **P < 0.01; ns, not significant; Student’s t test or Mann–Whitney U test as appropriate

We have previously identified genetic variants of Hippo pathway genes (YAP1 rs11225163, TEAD1 rs7944031 and TEAD4 rs1990330) and revealed that Hippo effector TAZ significantly associated with unfavorable survival in cutaneous melanoma and primary OSCC [7, 27, 28]. Given the increasingly appreciated roles of TEADs during tumorigenesis, we initially sought to explore the mRNA expression patterns of TEAD 1–4 in HNSCC using the publicly available TCGA dataset. As shown in Fig. 1a–d, TEAD2 and 4 were significantly upregulated in HNSCC samples as compared to their non-tumor counterparts, while TEAD1 and TEAD3 were markedly downregulated in cancerous samples relative to non-tumor samples. Moreover, four independent HNSCC patients cohorts from Oncomine database such as Peng’s [29], Ginos’ [30], Cromer’s [31] and Ye’s [32] cohorts were identified and utilized to measure TEAD4 mRNA expression. As shown in Fig. 1e–h, significantly higher abundance of TEAD4 mRNA was observed in HNSCC samples compared to their non-tumor counterparts. Several lines of evidence have revealed that TEAD4 is frequently amplified and/or aberrantly overexpressed in multiple cancers and associates with unfavorable prognosis [14, 15, 33]. Data integration and interrogation using cBioPortal platform indicated that total frequency of TEAD4 genetic alteration in HNSCC was rare, less than 2.5% in total patients. To identify potential associations between TEAD4 mRNA expression and clinicopathological parameters, we compared its abundance among diverse subgroups based on pathological grade and clinical stage, respectively. However, as shown in Additional file 1: Figure S1, the abundance of TEAD4 mRNA was comparable without significant difference among different subgroups stratified by pathological grade and clinical stage. In addition, there was no significant associations between TEAD4 mRNA expression and patient overall survival in TCGA-HNSCC cohort, when the median value of TEAD4 mRNA was used as cutoff to stratify patients into low and high TEAD4-expressing subgroups (Additional file 1: Figure S1).

### Overexpression of TEAD4 correlates with aggressive clinicopathological parameters in HNSCC

To further determine expression pattern of TEAD4 in HNSCC, we next performed immunohistochemical staining of TEAD4 in 105 primary HNSCC samples. The detailed demographic and clinicopathological parameters of these patients were listed in Table 1. In brief, 63 males and 42 females were enrolled with mean age 59.5 years. The follow-up durations ranged from 6 to 78 months (mean 39.5 months). Until the last follow-up, 77 (73.3%) patients remained alive with disease-free, 5 (4.8%) still alive but with recurrences and/or cervical nodal metastases, 23 (21.9%) died due to post-surgical recurrence, metastases or other unrelated diseases.

**Table 1:** The associations between TEAD4 expression and multiple clinicopathological parameters in HNSCC samples

Clinicopathological parameters	Cases	TEAD4		*P*-values
		Low*	High	
Gender	105	55	50	
Male	63	33	30	> 0.9999
Female	42	22	20	
Age				
≤ 60	43	22	21	0.8453
> 60	62	33	29	
Smoking				
No	74	37	37	0.5232
Yes	31	18	13	
Alcohol use				
No	83	42	41	0.6319
Yes	22	13	9	
Tumor size				
T1–T2	74	42	32	0.2011
T3–T4	31	13	18	
Pathological grade				
I	58	37	21	*0.0340*
II	34	13	21	
III	13	5	8	
Cervical node metastasis				
N(0)	71	43	28	*0.0214*
N(+)	34	12	22	
Clinical stage				
I	19	11	8	*0.0397*
II	30	21	9	
III	25	8	17	
IV	31	15	16	

* Both of patients with low and negative TEAD4 staining are stratified into low TEAD4 category for simplicity. The number in italic indicate statistical significance with *P*-values less than 0.05

**Fig. 2:**
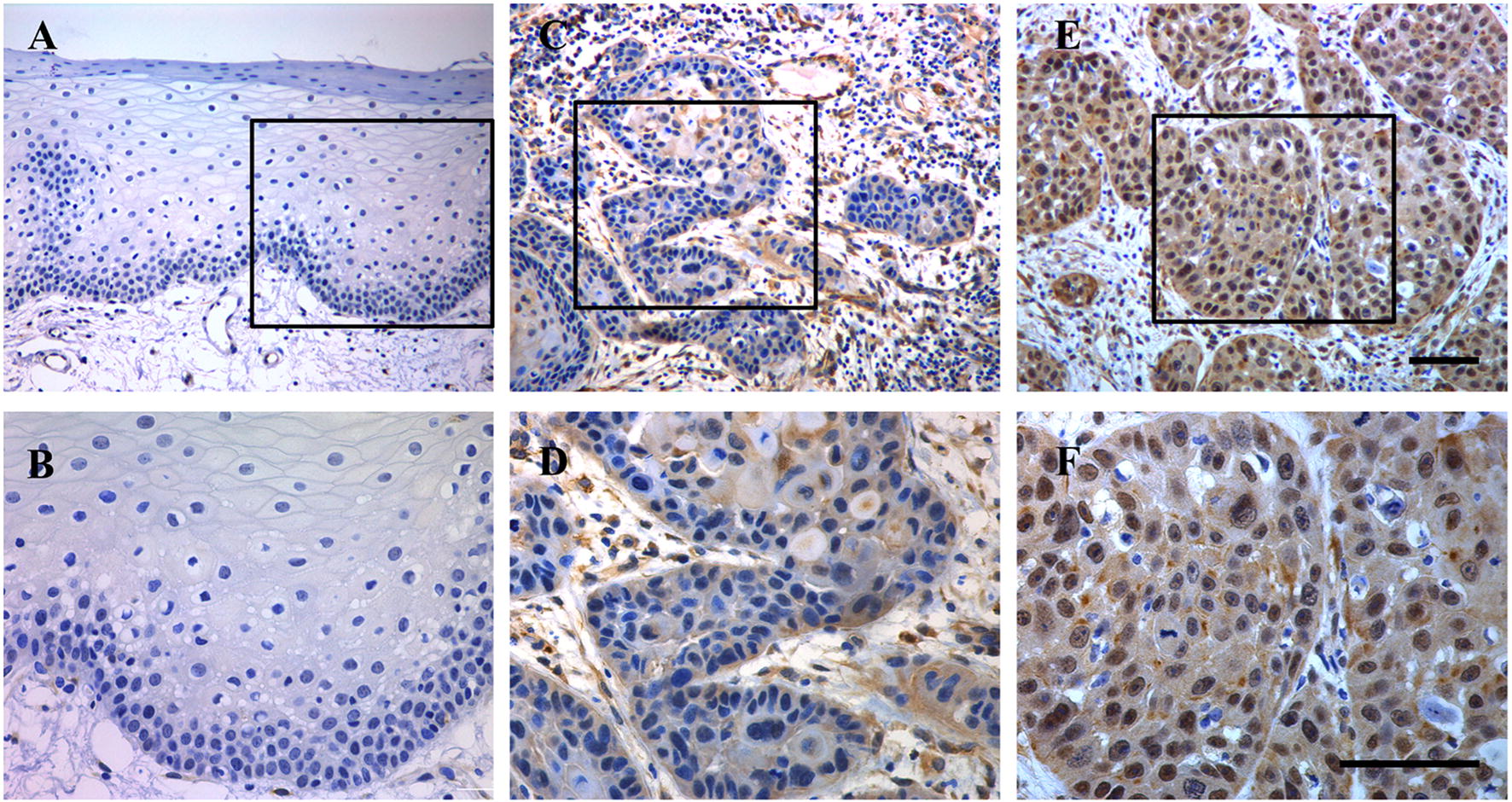
Immunohistochemical staining of TEAD4 in human HNSCC samples. **A**, **B** Representative negative staining of TEAD4 in normal oral epithelial; **C**, **D** representative low expression of TEAD4 in primary human HNSCC sample; **E**, **F** representative high expression of TEAD4 in primary human HNSCC sample. Nuclei are counterstained with hematoxylin. The areas marked by black box in the **A**, **C**, **E** images (upper panel) were shown in larger magnification as **B**, **D**, **F** images (lower panel), respectively. Scale bar: 100 μm

As shown in Fig. 2, rare positive staining of TEAD4 was observed in most healthy oral mucosa samples and a minority of HNSCC samples, whereas positive nuclear TEAD4 staining was detected in a fraction of HNSCC samples. TEAD4 expression patterns in HNSCC and normal oral epithelial were categorized according to immunohistochemistry scores. Consequently, TEAD4 protein abundance can be classified into low (55) or high expression (50) in HNSCC samples while negative (7), low (9) or high expression (4) in normal oral epithelial samples, indicative of aberrant TEAD4 overexpression in HNSCC (*P *< 0.0001, Chi square test). The detailed correlations between TEAD4 protein expression and clinicopathological parameters were further listed in Table 1. There was no significant associations between TEAD4 expression and gender, age, smoking, alcohol drinking and tumor size. However, high TEAD4 expression positively associated with advanced pathological grade, cervical node metastasis and advanced clinical stage with *P*-value 0.034, 0.0214 and 0.0397, respectively.

### Overexpression of TEAD4 significantly associated with reduced survival in HNSCC patients

**Fig. 3:**
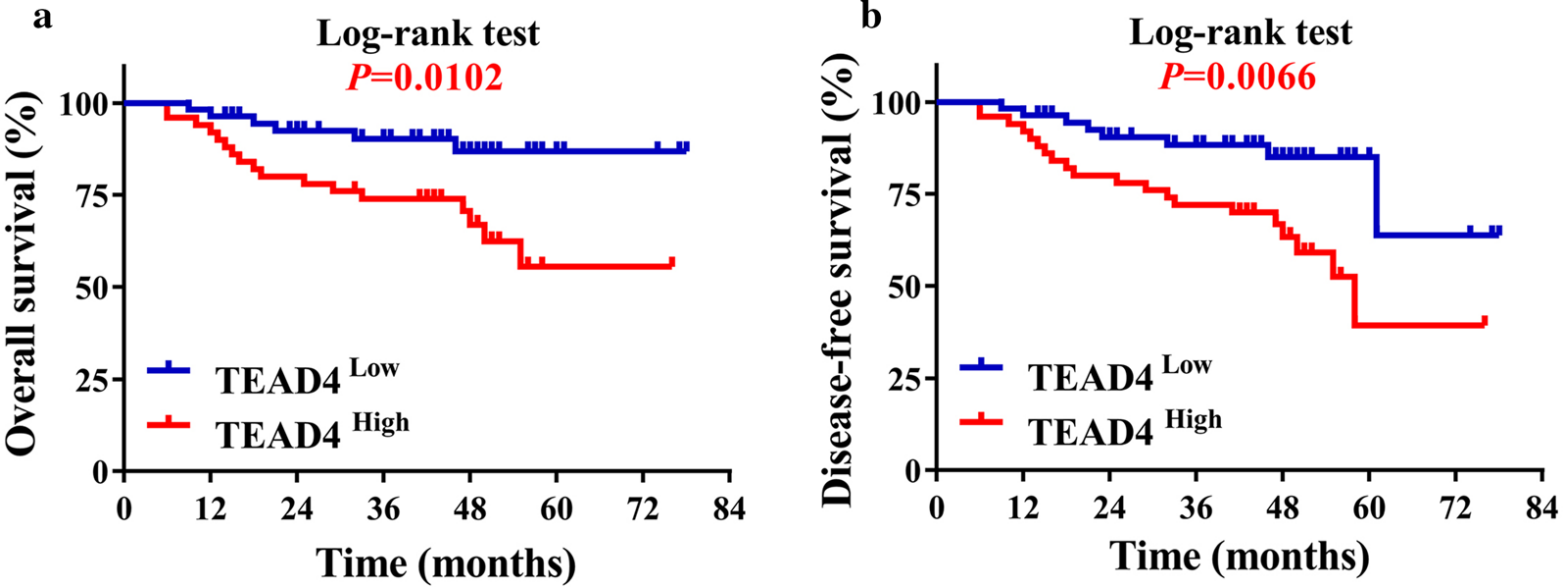
High TEAD4 expression positively associates with reduced overall survival in HNSCC patients. Overall survival (**a**) and disease-free survival (**b**) analyses of patients stratified with high or low expression of TEAD4 were estimated by Kaplan–Meier method and compared with Log-rank test

Next, we utilized Kaplan–Meier survival analyses to determine the association between TEAD4 expression and patient prognosis. As shown in Fig. 3, patients with high TEAD4 expression had significantly reduced overall survival and disease-free survival compared with those with low TEAD4 expression (log-rank test, *P *= 0.0102, 0.0066). Moreover, we applied both univariate and multivariate survival analyses to further evaluate the prognostic value of TEAD4 expression in HNSCC. In line with Kaplan–Meier survival analysis, the univariate regression assay revealed that TEAD4 expression was significantly associated with patient survival (*P *= 0.009). Furthermore, after adjusting for other demographic and clinicopathological parameters, TEAD4 expression was identified as an independent factor for patients’ survival (*P *= 0.028), along with the established prognostic factor, the clinical stage (*P *= 0.050, Table 2).

**Table 2:** Univariate and multivariate survival analyses (proportional hazards method) for patients with primary HNSCC

Variable	Univariate survival analysis			Multivariate survival analysis		
	Hazard ratio	95% CI	*P*-value	Hazard ratio	95% CI	*P*-value
Gender (male, female)	0.701	0.299–1.645	0.415			N/A
Smoking (No, Yes)	0.958	0.409–2.243	0.922			N/A
Alcohol use (No, Yes)	0.651	0.255–1.660	0.369			N/A
Age (≤ 60, > 60)	0.995	0.429–2.307	0.991	0.944	0.405–2.202	0.895
Tumorsize (T1–T2, T3–T4)	0.591	0.261–1.336	0.206	2.010	0.535–7.547	0.301
Pathological grade (I, II–III)	0.587	0.260–1.327	0.200	0.909	0.366–2.261	0.838
Cervical nodal metastasis (N0, N +)	0.540	0.238–1.225	0.140	1.816	0.483–6.832	0.377
Clinical stage (I–II, III–IV)	0.311	0.123–0.788	*0.014*	0.194	0.038–0.998	*0.050*
TEAD4 expression (low, high)	0.029	0.115–0.732	*0.009*	0.344	0.133–0.893	*0.028*

The numbers in italic indicate statistical significance with *P*-values less than 0.05

### Increased TEAD4 expression in chemical-induced HNSCC tumorigenesis

**Fig. 4:**
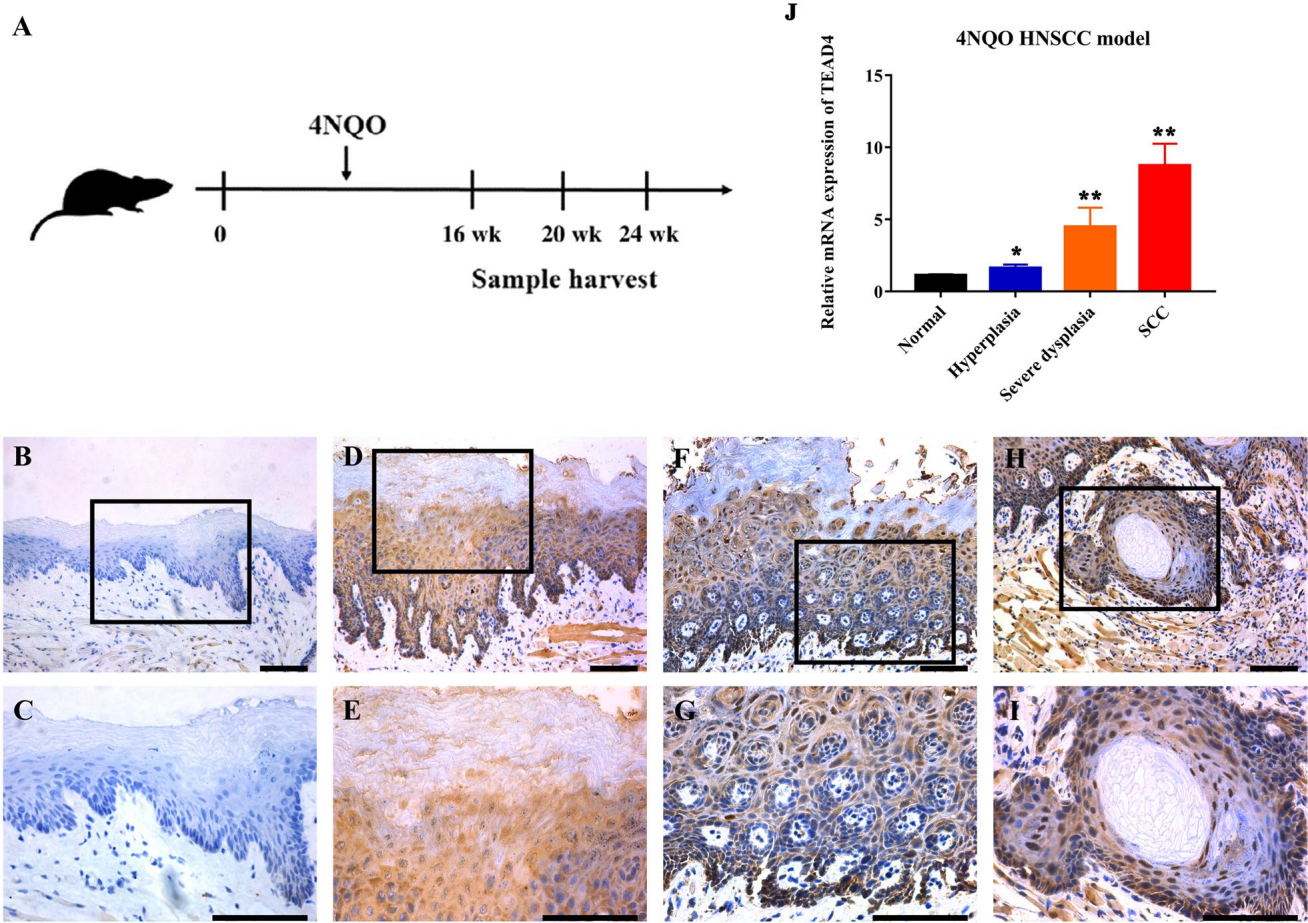
TEAD4 expression pattern during HNSCC tumorigenesis in 4NQO-induced animal model. **A** Experimental scheme of 4NQO-induced HNSCC animal model. **B**–**I** Immunohistochemical staining of TEAD4 in samples from diverse stages in 4NQO-induced animal model. Images in the upper panel (**B**, **D**, **F**, **H**) were representative staining of TEAD4 in normal, epithelial with hyperplasia, epithelial with severe dysplasia/carcinoma in situ and squamous cell carcinoma, respectively. Images in the lower panel (**C**, **E**, **G**, **I**) were magnified from the black box area in the **B**, **D**, **F**, **H** images in the upper panel, respectively. Scale bar: 100 μm. **J** The mRNA levels of TEAD4 during the 4NQO-induced HNSCC were measured by qRT-PCR in pre-stored samples (n = 5, 6 samples per group). *P < 0.05, **P < 0.01, ANOVA analyses

Having revealed aberrant overexpression of TEAD4 in a fraction of HNSCC, we wondered whether it played a role during HNSCC development. To address this, we utilized a well-established chemical-induced HNSCC animal model to characterize the expression pattern during HNSCC initiation and progression (Fig. 4A). In this model, lesions like hyperplasia, dysplasia, carcinoma in situ and invasive SCC were routinely found in the tongue, which resembles the pathological process of HNSCC. Thus, whole tongue was harvested at 16th, 20th and 24th week after 4NQO administration, which was subjected to histological analysis. As shown in Fig. 4B–I, immunohistochemical staining in these samples indicated significant strong nuclear staining of TEAD4 in carcinoma in situ and invasive carcinoma, while negative or low staining in normal tongue mucosa and epithelial with hyperplasia. Positive TEAD4 staining was commonly observed in carcinoma (87.5%, 7/8), but much less in samples with healthy mucosa (12.5%, 1/8), hyperplasia (25.0%, 2/8) or dysplasia/carcinoma in situ (37.5%, 3/8). Moreover, in line with the IHC findings, we found that the mRNA levels of TEAD4 in pre-stored carcinoma samples were significantly upregulated by qRT-PCR (Fig. 4J). Collectively, our findings from this well-established chemical-induced HNSCC model indicated that TEAD4 might serve as a putative oncogene driving HNSCC development.

### TEAD4 promotes cell proliferation, migration, invasion and EMT in HNSCC cells

**Fig. 5:**
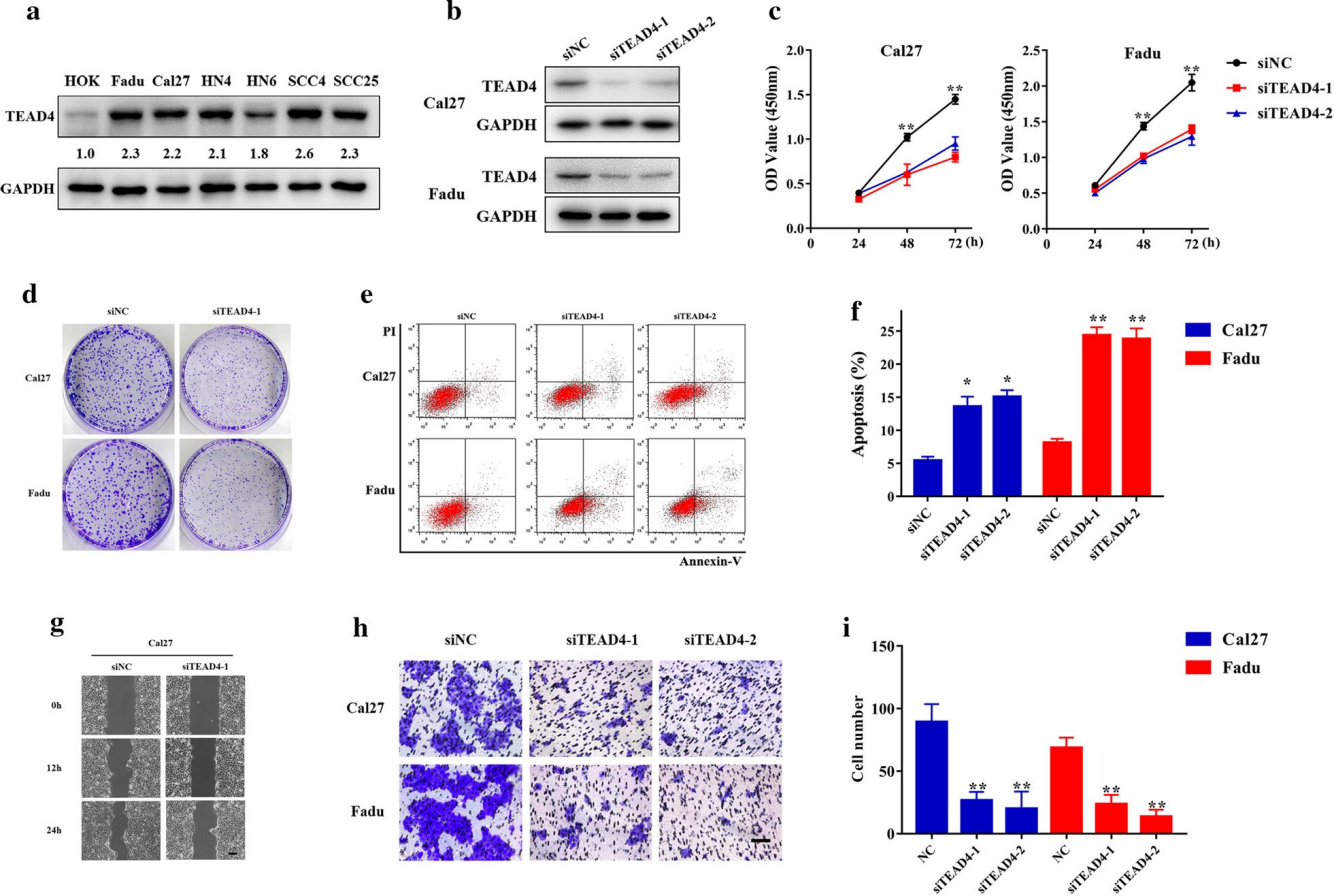
TEAD4 knockdown inhibits cell proliferation, migration and invasion, and triggers apoptosis in HNSCC cells. **a** Endogenous TEAD4 protein expression was measured in a panel of HNSCC cell lines as compared to normal oral epithelial (HOK). Representative images of western blot (WB) were shown from 3 independent experiments. **b** Endogenous TEAD4 was efficiently silenced by 2 siRNAs (siTEAD4–1, siTEAD4–2) in Cal27 and Fadu cells. Non-targeting siRNA was utilized as negative control (siNC). Representative images of WB are shown from 3 independent experiments. **c** Cell proliferation was remarkably suppressed when endogenous TEAD4 was silenced as measured by CCK-8 viability assay. **d** The potentials of colony formation were significantly inhibited in TEAD4-depleted cells (transfected with siTEAD4–1) as compared to control (siNC). **e**, **f** Increased percentages of cell undergoing apoptosis were evident following TEAD4 knockdown as assayed by Annexin V-PI staining. **g**–**i** The migration (**g**) and invasion (**h**) abilities were significantly reduced in siTEAD4-transfected cells in wound healing (12, 24 h after cell scratching) and transwell assays (12 h after cell seeding), respectively. Scale bar: 100 μm. Quantitive data of transwell assays were shown in **i**. Data shown here are mean ± SD from three independent experiments, *P < 0.05, **P < 0.01, ANOVA analyses with Tukey’s multiple comparisons test

Considering that our clinical results supported a potential pro-tumorigenic role of TEAD4 in HNSCC, we next aimed to delineate its oncogenic roles driving HNSCC initiation and progression by siRNA-mediated loss of-function approach. To address this, we first measured the abundance of TEAD4 in a panel of HNSCC cell lines and found that TEAD4 protein was significantly overexpressed in all HNSCC cell lines examined compared to immortalized oral epithelial cell (HOK) (Fig. 5a). Due to the relatively higher endogenous TEAD4 in Cal27 and Fadu cells, we next selected them for knockdown experiments. After 2 independent siRNAs targeting human TEAD4 were introduced into Cal27 and Fadu cells, the consequent changes of TEAD4 expression and cell phenotype were monitored. As shown in Fig. 5b, TEAD4 protein was significantly reduced in Cal27 and Fadu cells following siRNA transfection. Both results from CCK-8 viability assay and colony formation assay showed significantly lower proliferation rate upon TEAD4-siRNA transfection (Fig. 5c, d). Additionally, annexin V-PI flow cytometric assay revealed that the proportions of apoptotic cells in siTEAD4-treated cells were significantly increased from 5.5 to 15.1% in Cal27, from 7.6 to 24.4% in Fadu, respectively (Fig. 5e, f). Furthermore, the migratory and invasive potentials of cells were also measured using wound healing and transwell invasion assay after TEAD4 knockdown, respectively. As shown in Fig. 5g, h, the migratory and invasive abilities of cells following TEAD4 knockdown were significantly reduced.

**Fig. 6:**
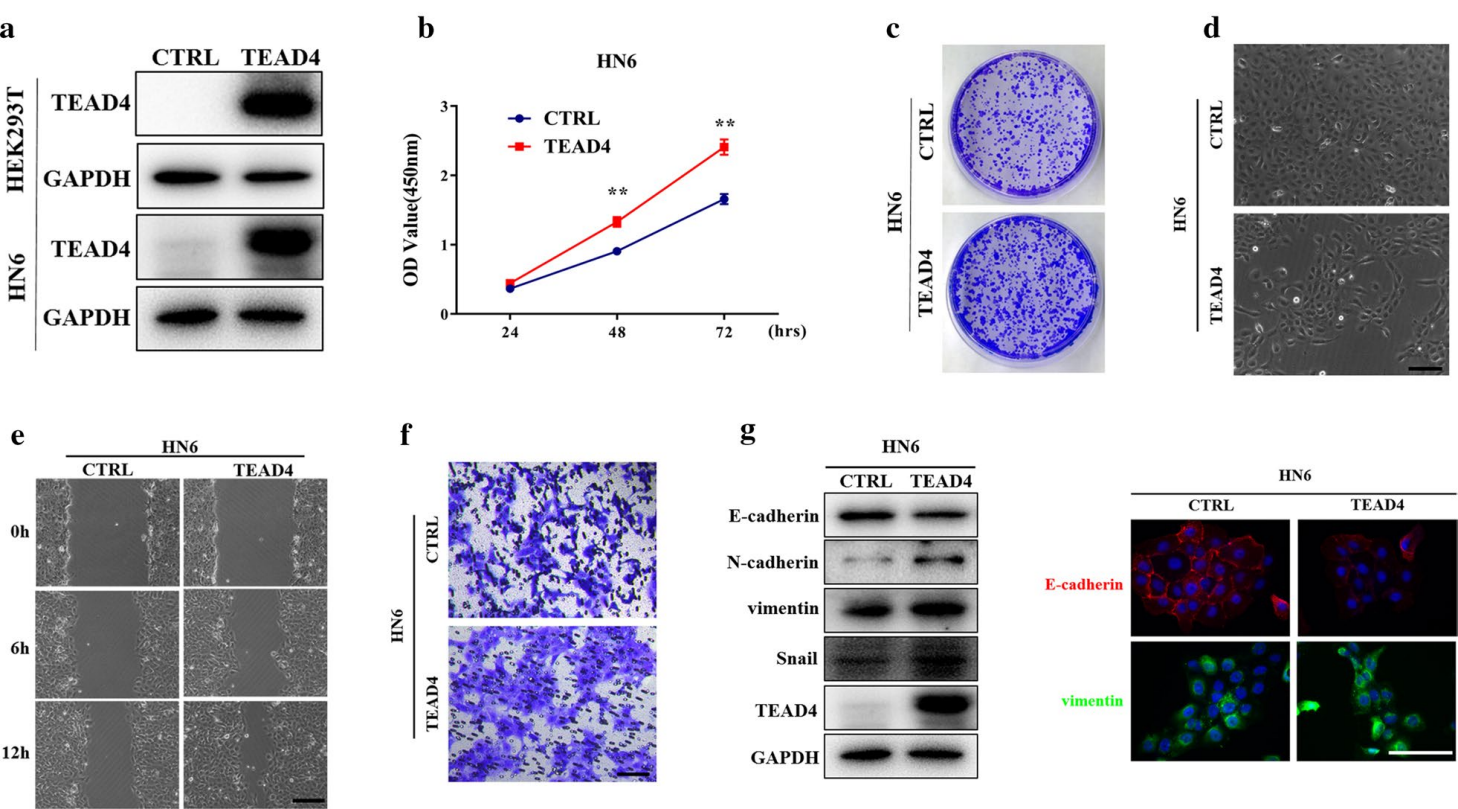
Ectopic TEAD4 overexpression induces EMT-like changes in HNSCC cells. **a** TEAD4 overexpression was confirmed by western blot in cellular lysates from 293T and HN6 cells infected with TEAD4 cDNA plasmid. Representative images of western blot (WB) were shown from 3 independent experiments. **b** Cell proliferation was remarkably promoted following TEAD4 overexpression by CCK-8 viability assay. **c** The potentials of colony formation were significantly promoted in TEAD4-overexpressed cells as compared to control. **d** Enforced TEAD4 overexpression resulted in EMT-like morphological changes from cobble-like to spindle-like appearance under phase contrast microscopy. **e**, **f** The cell motilities and invasion were remarkably enhanced after TEAD4 overexpression as gauged by wound healing (**e**) and transwell-invasion assay (**f**). Measurements of wound healing was performed at 6 and 12 h after cell scratching while measurements of transwell assays were done at 12 h after cell seeding. **g** The abundance of EMT markers E-cadherin, N-cadherin, Vimentin and Snail following TEAD4 overexpression were assayed by western blot (left panel). E-cadherin and vimentin expression was probed by immunofluorescent staining in TEAD4-overexpressed and control cells (right panel). Scale bar: 100 μm. Representative images are shown. Data showed here are mean ± SD from three independent experiments. **P < 0.01, Student-*t* test

To further confirm the tumorigenic roles of TEAD4 in HNSCC, we subcloned the full-length human TEAD4 cDNA with an N-terminal Flag tag into plasmid and generated stable TEAD4 overexpressing cells in HN6 (relatively low endogenous TEAD4) after antibiotics selection. As expected, TEAD4 protein was significantly increased in HN6 and HEK293T cells following plasmid transfection (Fig. 6a). We next utilized the HN6 cells with stable TEAD4 overexpression for subsequent experiments. Overexpression of TEAD4 accelerated cell proliferation in HN6 (Fig. 6b, c) as evidenced by results from CCK-8 and colony formation assay, which was consistent with the well-established pro-proliferative function of TEAD4. Interestingly, as shown in Fig. 6d, TEAD4-overexpressing cells became elongated, scattering distributed with fibroblast-like appearance, while control cells remained typical cobblestone morphology with tight adhesion. Moreover, remarkably enhanced migration and invasion abilities were observed in TEAD4-overexpressing cells (Fig. 6e, f). These morphological and functional changes induced by TEAD4 suggested that TEAD4 might be capable of promoting EMT in HNSCC cells. To confirm this notion, we further determined the levels of EMT-related markers by western blot and immunofluorescence assays. As shown in Fig. 6g, h, downregulation of E-cadherin and upregulation of N-cadherin, Vimentin and Snail were detected upon TEAD4 overexpression, which strongly suggested EMT following TEAD4 introduction in vitro.

### TEAD4 is involved in TGF-β1-induced EMT in HNSCC

**Fig. 7:**
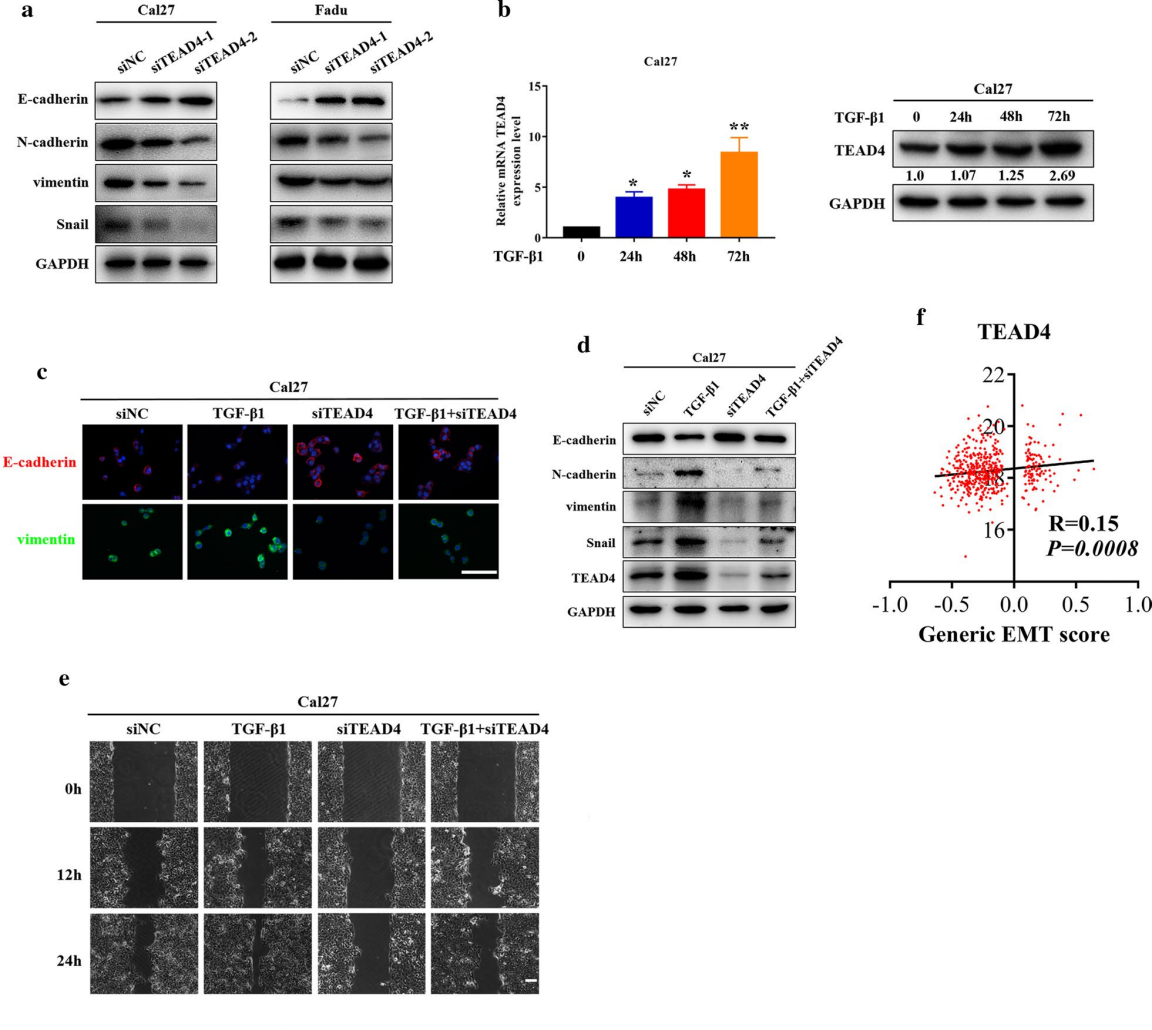
TEAD4 is required for TGF-β1-induced EMT in HNSCC cells. **a** The abundance of EMT-related markers E-cadherin, N-cadherin, vimentin and snail were measured by western blot (WB) in the Cal27 and Fadu cells following TEAD4 knockdown. **b** The mRNA and protein abundance of TEAD4 was measured by real-time RT-PCR and western blot when cells were treated with human recombinant TGF-β1 (10 ng/ml) at indicated time points. **c** The abundance of EMT-related markers E-cadherin, N-cadherin, vimentin and Snail were measured by immunofluorescence (**c**) and western blot assays (**d**) when Cal27 cells were treated siTEAD4 or in combination with TGF-β1 (10 ng/ml) for 48 h. **e** Cell migration was measured via wound healing assay when Cal27 cells were treated siTEAD4 or in combination with TGF-β1 (10 ng/ml) for 48 h. Scale bar: 100 μm. **f** Correlation between generic EMT score for 502 HNSCC samples from TCGA dataset and TEAD4 expression. Generic EMT score was calculated following the method of single-sample Gene Set Enrichment Analysis (ssGSEA) [36]. The correlation coefficient (R) and *P*-value were based on Pearson’s product-moment correlation analysis; R = 0.15, *P *= 0.0008. *P < 0.05, **P < 0.01, ANOVA analyses

Accumulating evidence indicates that EMT-mediated metastatic spread dictates patients survival in various solid cancers including HNSCC [34]. Both gain-of-function and loss-of-function in vitro assays suggested the potential roles of TEAD4 involving EMT and invasion of HNSCC. Thus, we next aimed to further verify the EMT-inducing role of TEAD4 in HNSCC. To address this, we firstly utilized two independent siRNAs to knockdown endogenous TEAD4 and revealed upregulation of E-cadherin and downregulation of Vimentin, N-cadherin and Snail following TEAD4 silencing in Cal27 and Fadu cells, which was well consistent with EMT-mediated marker changes (Fig. 7a). Next, we employed the well-established TGF-β1-induced EMT cell model [35] and found that both TEAD4 mRNA and protein expression significantly increased with TGF-β1-treated time in Cal27 (Fig. 7b). In addition, when cells were treated siTEAD4 alone or together with TGF-β1 for 48 h, immunofluorescence assay indicated that TGF-β1 exposure resulted in more cells with positive vimentin staining but less with positive E-cadherin staining, and TEAD4 knockdown by siRNA largely abolished these effects of TGF-β1 in vitro (Fig. 7c). Moreover, TGF-β1-induced EMT marker changes as measured by western blot assay were largely abolished upon TEAD4 depletion (Fig. 7d). In line with these marker changes, TGF-β1-induced enhancement of invasiveness were significantly impaired following TEAD4 knockdown (Fig. 7e). Finally, we extracted the original HNSCC dataset from TCGA platform and applied a generic gene signature of EMT to score the EMT status of these samples which was a versatile tool for objective and systematic investigation of EMT roles and dynamics in cancer progression [36]. Our results from EMT scoring revealed that TEAD4 expression was positively correlated with EMT score in HNSCC (Fig. 7f). Collectively, these results supported the critical roles of TEAD4 involved in TGF-β1-induced EMT in HNSCC, although the underlying regulatory network still required further delineation.

## Discussion

Until now, deregulated Hippo signaling pathway has been demonstrated to be intricately associated with tumorigenesis and serves as viable therapeutic targets with translational potentials [6]. TEAD4 functions as a key member of Hippo signaling mediating transcriptional output via forming complex with YAP or TAZ. Several lines with evidence have revealed that TEAD4 has oncogenic roles and prognostic significance underlying multiple cancer contexts [14, 15, 37, 39]. In the present study, we utilized the HNSCC samples, animal model and in vitro cellular assay to delineate the expression pattern, prognostic roles and tumorigenic functions of TEAD4 in HNSCC. Our findings revealed that TEAD4 served as a novel putative oncogene to promote HNSCC tumorigenesis and as a novel prognsotic biomarker for HNSCC.

Previous studies have revealed that TEAD4 is usually amplificated and/or overexpressed in multiple cancers including atypical teratoid/rhabdoid tumor, serous ovarian carcinoma, colorectal cancer, lung adenocarcinoma, gastric cancer and OSCC [14, 15, 33, 38–40]. In line with this, both bioinformatics analyses from multple independent patient cohorts and immunohistochemistry in primary HNSCC samples revealed aberrant overexpression of TEAD4 in a large subset of patients examined. Moreover, results from 4NQO-induced HNSCC model showed that TEAD4 expression increased along with disease initiation and progression from hyperplasia to invasive carcinoma. Although TEAD4 amplification was identified in selected cancers [33, 38], genetic amplification of TEAD4 was not prominent as evidenced by the fact that less than 2.5% HNSCC samples harbored genetic alternations of TEAD4, thus largely precluding the possibility of genetic amplification of TEAD4 responsible for its overexpression in most HNSCC samples. Together, these findings gave strong support to the idea that TEAD4 as a bona fide oncogene promotes HNSCC tumorigenesis, although the precise molecular mechanisms underlying its overexpression await further elucidation. To the best of our knowledge, this might be the first study to reveal the abnormal overexpression pattern of TEAD4 in HNSCC.

Several previous reports have proposed important clinical relevance of TEAD4 overexpression in human cancer [14, 15, 39]. For example, elevated TEAD4 expression significantly associated with advanced stage, distant metastasis and poor outcome in colorectal cancer [14]. Consistantly, our data from primary HNSCC samples revealed that TEAD4 overexpression significantly associated with high pathological grade, cervical node metastasis and advanced clinical stage. Moreover, results from Kaplan–Meier survival and univariate/multivariate Cox-regression analyses showed that TEAD4 overexpression significantly associated with reduced survival and served as an independent prognostic predictor for patients’ survival. However, we failed to reveal positive correlations between TEAD4 mRNA level and clinical grades, pathological stages as well as overall survival from TCGA-HNSCC dataset. We reasoned that it’s conceivable due to prominent heterogeneity of HNSCC and different strategies for patient stratification between TCGA-HNSCC cohort and our cohort. Inconsistency between mRNA and protein expression of TEAD4 might also account for this discrepancy. Of course, larger amount of patients with HNSCC from multiple centers is needed to establish the prognostic significance of TEAD4 and its clinical benefits of TEAD4 as a novel biomarker for patient stratification.

Previous reports have demonstrated that TEAD4 is critically involved in tumorigenesis by promoting cell proliferation, metastasis, EMT and suppressing apoptosis [14, 15, 37, 40]. For example, impaired cell proliferation and induction of G1 cell cycle arrest were observed in OSCC cell upon TEAD4 knockdown [40]. TEAD4 silencing markedly attenuated cell migration and invasiveness in lung adenocarcinoma [41]. In addition, increased nuclear TEAD4 expression promoted EMT and metastasis in colorectal cancer while its knockdown induced mesenchymal-epithelial transition and decreased cell mobility in vitro and metastasis in vivo [14]. Consistent with these above-mentioned findings, our results indicate that TEAD4 has multiple tumorigenic roles by modulating cell proliferation, apoptosis, migration and invasion in HNSCC cells. Noticeably, our results also indicated that TEAD4 promoted invasion and motility by facilitating EMT in HNSCC as evidenced by morphological alternations and EMT marker changes upon TEAD4 depletion and overexpression, its critical role during TGF-β1-induced EMT as well as positive association between TEAD4 expression and cervical node metastasis. Multiple downstream targets including vimentin and FSCN1 have been identified to mediate EMT induced by TEAD4 in colorectal cancer and gastric cancer [14, 42]. Moreover, Liu et al. [43] have reported that TEAD4 and AP1 co-occupy on active enhancer or promoter and drive a core set of downstream targets like CDH2 (encoding N-cadherin) to coordinate cancer cell migration and invasion. However, the downstream targets responsible for TEAD4-induced EMT in HNSCC remains unknown and requires further exploration. In addition, further studies are still needed to unravel the intricate crosstalk between TEAD4 and TGF-β pathway behind EMT and metastasis in HNSCC. Collectively, our findings together with others strongly suggest that TEAD4 probably functions as a putative pro-tumorigenic gene via enhancing cancer cell proliferation, migration and invasion in HNSCC.

## Conclusion

In conclusion, our findings revealed the expression pattern, prognostic and tumorigenic roles of TEAD4 and identified TEAD4 as a novel biomarker with diagnostic and prognostic significance in HNSCC and as a putative oncogenic mediator underlying HNSCC initiation and progression. Our findings suggest that selective targeting of TEAD4 by genetic or chemical approach might hold translational promise against HNSCC.

## Additional file

**Additional file 1: Figure S1.** A: Relative expression of TEAD4 mRNA (Log2-transformed) was compared TCGA-HNSCC subgroups stratified by pathological grades. NS denotes not significant difference between groups. Y-axis represents the median intensity, 25th, and 75th percentile data. B: Relative expression of TEAD4 mRNA (Log2-transformed) was compared in TCGA-HNSCC subgroups stratified by clinical stage. Y-axis represents the median intensity, 25th, and 75th percentile data. C: Overall survival analyses of TCGA-HNSCC patients with high or low expression of TEAD4 mRNA (median value as cutoff) were estimated by Kaplan-Meier method and compared with Log-rank test.

## Abbreviations

HNSCC: head neck squamous cell carcinoma
TEADs: the TEAD transcription factor
4NQO: 4-nitroquinoline 1-oxide-induced HNSCC animal model
EMT: epithelial-mesenchymal transition
