# Supplementary figures and images for: TEAD4 overexpression promotes epithelial-mesenchymal transition and associates with aggressiveness and adverse prognosis in head neck squamous cell carcinoma
Source: Cancer Cell Int. 2018 Nov 12;18:178. doi: 10.1186/s12935-018-0675-z (PMC6233371; doi:10.1186/s12935-018-0675-z)

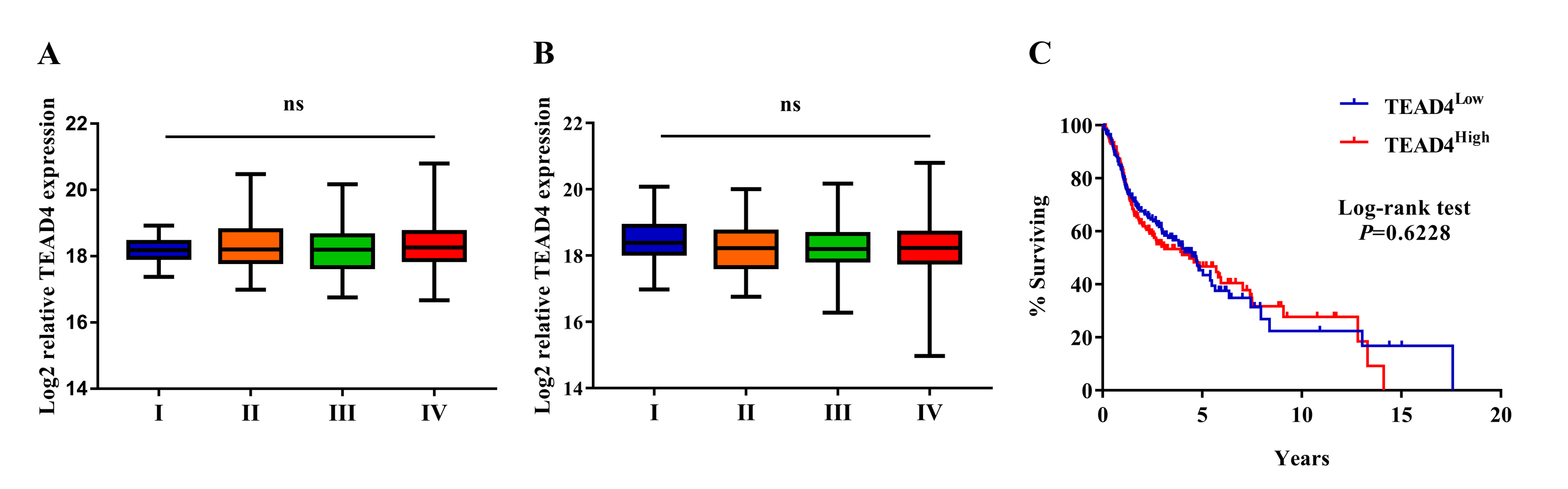

Supplement: Supplementary file 1 — Additional file 1: Figure S1. A: Relative expression of TEAD4 mRNA (Log2-transformed) was compared TCGA-HNSCC subgroups stratified by pathological grades. NS denotes not significant difference between groups. Y-axis represents the median intensity, 25th, and 75th percentile data. B: Relative expression of TEAD4 mRNA (Log2-transformed) was compared in TCGA-HNSCC subgroups stratified by clinical stage. Y-axis represents the median intensity, 25th, and 75th percentile data. C: Overall survival analyses of TCGA-HNSCC patients with high or low expression of TEAD4 mRNA (median value as cutoff) were estimated by Kaplan-Meier method and compared with Log-rank test. [file 12935_2018_675_MOESM1_ESM.jpg]
